# Optical brain imaging and its application to neurofeedback

**DOI:** 10.1016/j.nicl.2021.102577

**Published:** 2021-01-26

**Authors:** Surjo R. Soekadar, Simon H. Kohl, Masahito Mihara, Alexander von Lühmann

**Affiliations:** a Clinical Neurotechnology Laboratory, Dept. of Psychiatry and Psychotherapy, Neuroscience Research Center, Campus Charité Mitte (CCM), Charité – University Medicine of Berlin, Berlin, Germany; b JARA-Institute Molecular Neuroscience and Neuroimaging (INM-11), Jülich Research Centre, Jülich, Germany; c Child Neuropsychology Section, Department of Child and Adolescent Psychiatry, Psychosomatics and Psychotherapy, Medical Faculty, RWTH Aachen University, Germany; d Department of Neurology, Kawasaki Medical School, Kurashiki-City, Okayama, Japan; e Machine Learning Department, Computer Science, Technische Universität Berlin, Berlin, Germany; f Neurophotonics Center, Biomedical Engineering, Boston University, Boston, USA

**Keywords:** Optical brain imaging, Brain-computer interface, Functional near-infrared spectroscopy, Neurovascular coupling, Cerebral blood flow, Clinical translation, Neuropsychiatric disorders, Neurofeedback

## Abstract

Besides passive recording of brain electric or magnetic activity, also non-ionizing electromagnetic or optical radiation can be used for real-time brain imaging. Here, changes in the radiation’s absorption or scattering allow for continuous *in vivo* assessment of regional neurometabolic and neurovascular activity. Besides magnetic resonance imaging (MRI), over the last years, also functional near-infrared spectroscopy (*f*NIRS) was successfully established in real-time metabolic brain imaging. In contrast to MRI, *f*NIRS is portable and can be applied at bedside or in everyday life environments, e.g., to restore communication and movement. Here we provide a comprehensive overview of the history and state-of-the-art of real-time optical brain imaging with a special emphasis on its clinical use towards neurofeedback and brain-computer interface (BCI) applications. Besides pointing to the most critical challenges in clinical use, also novel approaches that combine real-time optical neuroimaging with other recording modalities (e.g. electro- or magnetoencephalography) are described, and their use in the context of neuroergonomics, neuroenhancement or neuroadaptive systems discussed.

## Highlights

• A comprehensive review of the SoA in real-time optical brain imaging is provided.
• Besides communication and movement, applications targeting cognition are introduced.
• Hybrid systems combining different modalities (e.g. EEG and *f*NIRS) are discussed.
• Merging assistive BCI and neurofeedback may boost efficacy and adoption.

## 1 Introduction

Imaging-based neurofeedback or brain-computer interface (BCI) applications depend on reliable real-time imaging of neural processes reflected by various physiological measures, such as single or multi-unit spike activity, electric or magnetic cortical field potentials, neuro-electric or -magnetic brain oscillations, or cerebral blood flow and oxygenation(Lebedev and Nicolelis, 2017, Soekadar et al., 2008). While some of these measures are recorded *passively*, i.e., by amplification of signal power originating from the brain itself (e.g., electric or magnetic field potentials and oscillations), others are assessed by directing energy to the brain, e.g., in the form of non-ionizing electromagnetic or optical radiation, and measuring its absorption, scattering, reflection or transmission. Here, due to their biological compatibility and tolerability, use of static magnetic fields, spatial and temporal gradients of magnetic fields, as well as optical radiation, e.g., in the near-infrared spectrum (700–2500 nm), was successfully established in brain imaging over the last years. Being portable and broadly accessible, particularly functional near-infrared spectroscopy (*f*NIRS) developed into a promising tool in neurofeedback and BCI applications (Naseer & Hong, 2015). Although sharing very similar technical underpinnings, the distinction between neurofeedback and BCI applications mainly derives from their purpose of use: while the term neurofeedback was introduced to denote techniques facilitating self-regulation of brain/neural activity through sensory feedback, e.g., to normalize symptom-related brain activity, BCI is typically used to label technical tools that provide active brain/neural-control of external devices, e.g., for restoration of communication or movement (Birbaumer et al., 2014, Nannet al., 2020, Soekadar et al., 2008, Soekadaret al., 2016, Wolpawet al., 2002). Beside such *active* BCIs, also paradigms in which brain/neural activity informs human–machine interaction passively became included under the term BCI (then termed *passive* BCI or neuroadaptive technology) (Blankertzet al., 2010, Klaprothet al., 2020, Mulleret al., 2008, Zander and Jatzev, 2012, Zander and Kothe, 2011), e.g., when adjusting user interfaces in the operational theater to the surgeon’s mental state (Arico et al., 2017). As such, most BCI systems feature qualities of neurofeedback, and it was proposed that merging these two different types of application, e.g. in the context of neurorehabilitation (Soekadaret al., 2015a), may boost efficacy and adoption of clinical BCIs (Soekadar et al., 2019).

Being noninvasive and wearable, real-time optical brain imaging is a promising modality for both neurofeedback and BCI applications. To appreciate its advantages and recent technical innovations on one side and discuss current challenges and limitations on the other side, we first provide a comprehensive overview of the history and state-of-the-art in optical brain imaging, before highlighting the most recent studies on its application to brain/neural control and neurofeedback.

## 2 Use of optical radiation to assess brain physiology

When measuring the heating effects of sunlight at different wavelengths, Fredrick William Herschel (1792–1871) discovered that this heating effect increased from the blue to the red part of the optical spectrum and that the thermal effects even further continued beyond the visible spectrum so that he postulated the existence of invisible “calorific rays” (Herschel, 1800). Later, this part of the optical spectrum was termed infrared radiation (spectral wavelength 700–1000 nm).

Based on the discovery that specific elements generate characteristic absorption-lines when exposed to optic radiation (Fraunhofer, 1817), near-infrared light was later used, for instance, in the context of spectrochemical analysis (Abney & Festing, 1881). With the development of very sensitive light detectors (such as photomultiplier tubes or charge-coupled device sensors) and fiber-optic light guides, a whole set of applications in physics, chemistry, and bio-physiology emerged that was based on inferring molecular properties from attenuation of optical radiation.

Oxygenation of hemoglobin alters its absorption characteristics within the near-infrared spectral band between 784 and 894 nm (Hoppe-Seyler, 1864, Stokes, 1864). As biological tissue, including the skull, is partially transparent to optical radiation at wavelengths in the (near-)infrared spectral band between 700 and 1000 nm, near-infrared radiation is particularly attractive to assess, e.g., brain metabolic processes associated with a modification of light absorption and scattering (Jobsis, 1977). Using the Beer-Lambert law (Beer, 1852), the absorbance and concentration in a sample can be related to the attenuation of light with known wavelength and intensity that irradiates this sample. In 1988, Delpy provided a modification of the Beer-Lambert law by taking light scattering into account, e.g. under the condition that it was kept constant and stable. This so-called ”modified Beer-Lambert law” permitted the calculation of relative oxygenation levels from the measured near-infrared spectroscopy (NIRS) signal (Delpy et al., 1988) (a technique termed *functional* NIRS or *f*NIRS when used for the purpose of assessing metabolic responses linked to a specific function or behavior). Given that attenuation of near-infrared radiation passing through a specific medium relates to the concentration of light-absorbing molecules (chromophores) in the medium, e.g. oxygenated and deoxygenated hemoglobin (oxy-Hb and deoxy-Hb), changes in molecule concentration can be calculated. This derives from the modified Beer-Lambert law with the following equation:

$$\Delta A\left(\Delta t,\lambda \right)=\sum_{i}\varepsilon_{i}\left(\lambda \right)\Delta c_{i}P\left(\lambda \right)d$$

where Δ*A* is the measured change in attenuation of light within two consecutive time points *Δt,* the index *i* denotes all investigated chromophores, *ε_i_* is the corresponding extinction coefficient at wavelength λ, Δ*c_i_* is the change in concentration, *d* is the source-detector separation, and *P* is the differential path length factor that accounts for increased distance traveled by the light due to scattering in the tissue. Provided the scattering/path length *d* × *P* are known, absolute changes in the concentration of a specific chromophore can be calculated. This path length can be estimated using three different approaches: 1. By assessing the phase shift of an intensity-modulated light source, 2. by measuring the direct ‘time of flight’ of short light pulses passing through the substrate, and 3. by measuring absorption at wavelengths that are typical for water molecules assuming constant water concentrations throughout the medium.

There is ample evidence for a close relationship between local neuronal activity and dilation of nearby blood vessels as well as increase in local blood flow. The spatial and temporal correlation between transient modulations in neural activity and associated hemodynamic responses was termed neurovascular coupling (NVC) (Iadecola, 2017). Building on NVC, there are now three established optical methods to assess modulations of regional neurometabolic and neurovascular activity as surrogate marker for neural activity: 1. intrinsic signal optical imaging, 2. diffuse correlation spectroscopy (DCS) (Durduran & Yodh, 2014) and 3. *f*NIRS (Scholkmann et al., 2014). While intrinsic signal optical imaging offers high spatial (~100 μm) and temporal (100 ms) resolution, it requires open-skull or thinned-skull procedures and has only been established in animal research. DCS measures speckle fluctuations of near-infrared diffuse light (i.e., fluctuations in intensity patterns produced by the mutual interference of a set of optical wavefronts) in tissue that are sensitive to the motions of red blood cells. Offering high temporal resolution (up to 100 Hz), a relatively large penetration depth (up to ~1.5 cm) and portability, it is a promising new technique, but has not been established in the neurofeedback or BCI fields yet. Finally, *f*NIRS is the most established and fastest growing approach in neurofeedback/BCI applications, mainly because of its robustness, portability and commercial accessibility.

**Fig. 1:**
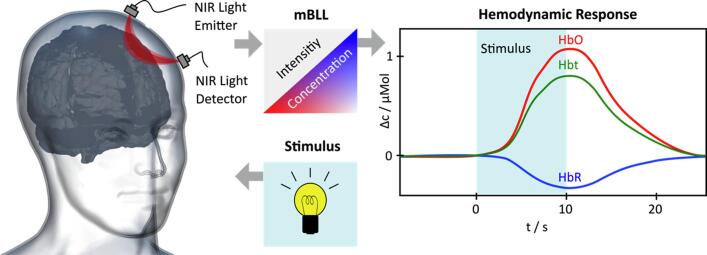
Assessing brain hemodynamic responses using functional near-infrared spectroscopy (*f*NIRS). Near-infrared (NIR) light (e.g., at 760 nm and 850 nm) is emitted through the subject’s skull (left panel). Changes in intensity of light absorption and scattering in response to a stimulus are continuously recorded by a NIR light detector. Based on the modified Beer-Lambert Law (mBLL), the measured light intensity can be converted into estimations of cerebral total hemoglobin (HbT) and differentiated into oxygenated and deoxygenated hemoglobin (HbO/HbR) (right panel, t = time, s = seconds, c = concentration).

All three techniques can evaluate relative changes of oxy- and deoxy-Hb or cerebral blood flow in superficial cortical layers. It was shown that neural activity resulting in higher oxygen consumption leads to decreased oxy-Hb concentrations (termed “initial dip”) (Bahar et al., 2006), followed by an up-regulation of regional cerebral blood flow (rCBF) within a few hundred milliseconds to seconds. This up-regulation is followed by an increase of total hemoglobin (t-Hb) that, overall, results in a measurable increase in oxy-Hb. As the degree of rCBF increase was shown to exceed the regional cerebral oxygen metabolic rate (rCMRO_2_) during neural activity (Fox & Raichle, 1986), neural activity can be estimated from the relative increase in oxy-Hb and t-Hb compared to a relative decrease of deoxy-Hb in the venous branch of the cerebral vascular system (Wolf et al., 2002) (Fig. 1).

It should be noted, however, that there is no clear mechanistic understanding yet of how neuronal activity regulates cerebral blood flow and metabolism as measured by, e.g., *f*NIRS or *f*MRI (Devor et al., 2012). According to the “metabolic hypothesis”, neural activity associated with an increase in lactate, adenosine triphosphate/adenosine diphosphate ratio or modulation of some yet unidentified oxygen sensor (Paulsonet al., 2010, Raichle and Mintun, 2006), for instance, leads to a metabolic cascade resulting in vasodilatation/constriction. An alternative hypothesis, the “neurogenic hypothesis”, postulates feed-forward mechanisms in which release of neurotransmitters and neuropeptides influence CBF and metabolism directly. It is conceivable that both mechanisms play a role, and that also glial cells contribute to these mechanisms (Allamanet al., 2011, Iadecola, 2017).

**Fig. 2:**
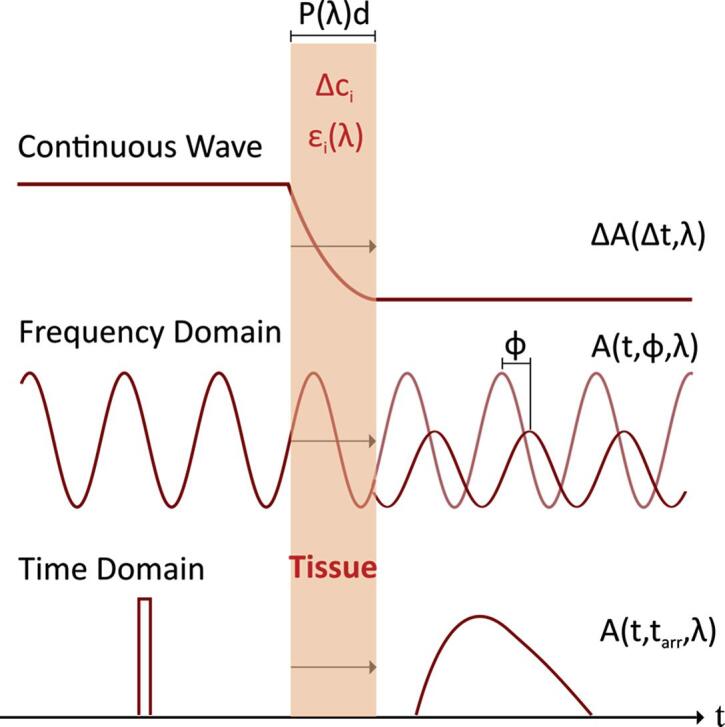
Most commonly used functional near-infrared spectroscopy (*f*NIRS) methods to assess molecular properties of organic tissue (modified from Scholkmann et al., 2014). The index i denotes all investigated chromophores, P is the differential path length factor that accounts for increased distance traveled by the light due to scattering in the tissue, and d is the source-detector separation. Δc*_i_* is the change in concentration and ε_i_ is the corresponding extinction coefficient at wavelength λ, ΔA is the measured change in attenuation of light within two consecutive time points Δt. φ is the estimated phase shift between the emitted and detected light waves. Currently, most BCI applications use continuous wave (CW) spectroscopy because it is particularly suitable to continuously assess relative changes in, e.g., oxy-/deoxy-hemoglobin.

Currently, there are three methods available to infer molecular properties of organic tissue using *f*NIRS: 1. continuous-wave (CW) spectroscopy, 2. frequency-domain (FD) techniques, and 3. time-domain (TD) (or time-resolved) spectroscopy (Fig. 2). State-of-the-art NIRS systems can comprise up to several hundred channels with temporal resolutions as high as 250 Hz and spatial resolutions of approximately 7–10 mm (Ferrari & Quaresima, 2012).

In CW spectroscopy, a continuous wave of light with either discrete wavelengths (laser diode) or sharply peaked/narrow spectra is used. Typically, continuous-wave NIRS sensitivity is increased by applying amplitude-modulation at low frequencies (in the kHz-range) and using phase-locked detection techniques. While the CW approach can achieve high signal-to-noise ratios, a major disadvantage is that CW spectroscopy is unable to quantify light scattering. Thus, light absorption and absolute hemoglobin concentrations can only be estimated based on relative changes, assuming constant scattering, not allowing for resolving contributions of different tissues. Here, to some degree, the use of multiple source-detector distances (M s-d) allows for separating different tissue layers and can substantially improve signal-to-noise ratio (Gagnonet al., 2012, Yucelet al., 2015).

Frequency-domain techniques also use a continuous wave, but modulate its intensity at high frequencies (100 MHz–1 GHz), using measured phase shifts for estimation of the time of flight. While potentially providing more precise recordings, the advantages of FD techniques over conventional CW techniques have yet to be established (Davies et al., 2017). In time-domain or time-resolved spectroscopy, picosecond light pulses are applied and the photon arrival times are measured directly. Besides determining molecule concentrations from absorption changes, these approaches also provide scattering information and thus enable the separation of different tissue layers. Recently, a first optical BCI was introduced based on such time-resolved *f*NIRS signal potentially applicable for mental communication in patients with brain injury or stroke (Abdalmalak et al., 2020).

As most neurofeedback and BCI paradigms utilize relative and not absolute changes of brain physiological parameters, up to now mainly CW spectroscopy was established in the neurofeedback/BCI field. FD and TD spectroscopy, however, may offer some complementary advantages that need to be further explored in real-time optical imaging. While CW spectroscopy is already widely available and can be more easily applied in multichannel systems due to its mobility (Ferrari & Quaresima, 2012), FD and TD techniques are potentially more precise due to their penetration depth (Coscia et al., 2019, Gagnonet al., 2012, Yucelet al., 2015), but also less established and more cost intensive.

The distance between CW *f*NIRS light sources and detectors is a tradeoff between signal strength (light attenuation and the pathlength of light are exponentially related) and sensitivity to brain tissue (for a larger source-detector separation, more photons ‘see’ the cerebral layer of interest). Injected light has to conform with safety standards and powers usually lie in the range of 5–50 mW. For transcranial assessment of human cortical hemoglobin oxygenation, the light sources and detectors are typically placed at a distance between 2 and 4 cm.

A remaining major challenge in using *f*NIRS to assess brain physiological activity is the differentiation between neurometabolic/neurovascular signals and other systemic biological processes. For instance, non-neural activity related to systemic arterial pulse oscillations (~1 Hz), respiration (~0.2 – 0.4 Hz) or and low frequency oscillations of other origin (at <~0.1 Hz) (*Mayer waves/Traube-Herring waves*) modulate the measured oxy-Hb and deoxy-Hb concentrations, and fluctuate both during task and at rest conditions (Toronov et al., 2000). Although a number of strategies were introduced to reduce the impact of these systemic physiological artifacts (Klein and Kranczioch, 2019, von Luhmannet al., 2020), validity of quantitative measures is still limited. Thus, most neurofeedback/BCI applications use a dual-state approach, i.e., (continuously) contrast two known conditions for the classification of brain states. Here, relative changes of oxy-Hb, deoxy-Hb and CBF are sufficient for simple brain-state assessment. Another limitation related to real-time monitoring of hemoglobin is the considerable delay between neural activation and change in oxy-Hb/deoxy-Hb (ranging from two to several seconds) (Huppert et al., 2006). While the change in oxy-Hb/deoxy-Hb during a simple motor activation paradigm, e.g. an externally-paced button press task, usually occurs at 2–5 s after task execution, experimental designs with an impact on systemic parameters (e.g. ventilation) can lead to a delayed change of oxy-Hb/deoxy-Hb at 1–4 min after the onset of the task. Here, the signal change occurs due to insufficient source separation between brain and other tissue, e.g. skin and connecting tissue, that leads to contamination of brain tissue-related measures. Thus, most neurofeedback and BCI applications apply a moving baseline to compensate for this issue.

With the advancement of optical imaging methods and translation from microscopic to macroscopic scales, other metabolic responses to neural activity (e.g., voltage shifts, ionic indicators or synaptic release) may become viable for non-invasive optical BCI applications. In this context, acousto-photonic tomography and photo-acoustic tomography (Yao and Wang, 2014) may play a particular role, but there are a number of challenges related to penetration depth, resolution and susceptibility to motion artifacts that have yet to be mastered.

## 3 Optical brain imaging and its application to neurofeedback

Given the variety of physiological parameters that can be derived from changes in optical properties of brain tissue (e.g., perturbations in cerebral blood volume, blood flow or metabolic rate of oxygen), several possible clinical *f*NIRS applications were explored over the last years (Mihara and Miyai, 2016, Naseer and Hong, 2015, Wolfet al., 2007). These range from studying the physiological correlates of stroke and cerebrovascular disease to epileptic disorders (e.g., oxygenation response to epileptic activity and focus localization), idiopathic headache syndromes and functional imaging of the diseased brain (e.g., in the context of neurorehabilitation) (Obrig, 2014). *f*NIRS also showed particular promise as a clinical tool for online brain-monitoring during cardiac surgery (Zheng et al., 2013) or critical care (Moerman & De Hert, 2017). It is worth noting that (Kannan and Przekwas, 2011, Kannan and Przekwas, 2012) have performed very high fidelity 2D and 3D simulations for accurately and efficiently predicting and quantifying local and global injuries for organs like the brain and the lung. They were able to (i) noninvasively “numerically penetrating” the tissues and (ii) reconstruct the optical properties the presence of water, oxygenated, and de-oxygenated blood. These numerical noninvasive measurements are then used to predict the extent and severity of the organ hemorrhage/injury. A major obstacle in establishing NIRS as a routine clinical tool for diagnostics of the diseased brain is the variety of NIRS parameters (such as change in cerebral blood volume, blood flow or oxy-Hb/deoxy-Hb), however, and variability of tasks that are used in clinical studies. This limits comparability and generalizability of findings. Currently, commercial systems provide information on relative change in hemodynamic properties only, but not on absolute change. Also, as NIRS can only assess the optical properties of the superficial cortical layers, deep sulci, as well as all subcortical and infra-tentorial brain regions cannot be studied directly. Moreover, compared to oxy-Hb/deoxy-Hb as measured by *f*NIRS, signal-to-noise of fMRI BOLD signals was estimated to be up to 2–3 times higher (Cui et al., 2011). While NIRS clearly has the advantage that it can be used at the patient’s bedside, its superiority over other diagnostic tools remains to be proven.

The first studies aiming at establishing *f*NIRS as a modality for brain self-regulation were performed at the beginning of the millennium (Coyleet al., 2004, Sitaramet al., 2005). It was demonstrated that motor imagery, e.g., the imagination of clenching a ball, is associated with a distinct oxy-Hb concentration increase and deoxy-Hb concentration decrease as measured by contralateral optodes placed over the motor cortex (C3/C4 electrode positions according to the 10/20 EEG system). This metabolic modulation was translated into online visual feedback, e.g., displayed as a growing or shrinking circle on a computer display (Coyle et al., 2004). In a later study, this paradigm was used to establish a binary switch. In this study, two options (e.g., “yes” and “no”) were presented to the user who was instructed to perform imagery tasks only when the desired target became highlighted (a control paradigm commonly referred to as “synchronous mode of operation”). The system monitored the sensorimotor cortex activity during both options and compared the response to each. The selection of either state required multiple trials and, after approximately one minute, the classification result was shown on a display. Although performance (defined as information transfer rate, ITR, in bit/sec.) was inferior to many electroencephalographic (EEG) systems, the system could achieve more than 80% correct classifications in healthy participants (Coyle, 2007), a value comparable with other established EEG- or magnetoencephalography (MEG)-based BCIs (Mihara & Miyai, 2016).

Given the tight relatedness of neurofeedback and BCI applications on their technological but also clinical level (Soekadar et al., 2019), the following provides an overview of the most relevant applications that employ real-time optical brain imaging, e.g., for restoration of communication and movement, symptom reduction in neuropsychiatric disorders, or neuroenhancement and neuroergonomics^1^. From a behavioural point of view, both neurofeedback and BCI applications involve operant conditioning of neural cell esemblies (Soekadar et al., 2015b). This shared neurobiological basis may not only potentiate the scope of BCI applications from assistance to treatment, but may also pave the way for the development of novel mechanism-based neurofeedback paradigms.

### 3.1 Optical BCIs in restoration of communication

The first clinically meaningful application of a BCI was introduced in the late 1990ies when a patient who suffered from locked-in syndrome (LIS) could select single letters on a screen by self-regulating slow cortical potentials (SCP) recorded by EEG. Applicability and versatility of such BCI systems for restoration of communication were later substantially improved (Blankertzet al., 2011, Blankertzet al., 2008, Kubler and Kotchoubey, 2007). However, successful application in patients diagnosed with *complete* locked-in syndrome (CLIS), i.e., the inability to elicit any voluntary muscle contraction, remains a great challenge. While not all reasons for this failure in applicability are entirely clear and remain subject of discussion, e.g., the *thought-extinction hypothesis* (Kubler & Birbaumer, 2008) predicting that any voluntary cognitive activity, goal-directed thinking or mental imagery would cease once a patient has entered CLIS, an in-depth analysis of data recorded by electrocorticography (ECoG) showed that CLIS can lead to considerable impairment of the circadian system reflected by an increased fragmentation of slow wave sleep (SWS) (Soekadar et al., 2013). This evidenced that use of appropriate tools to monitor attentiveness and alertness during the attempt to establish any form of BCI communication with CLIS patients is critical.

Assuming that instrumentally learned responses and intentional cognitive processes extinguish in complete paralysis (as predicted by the thought extinction hypothesis), alternative approaches based on *semantic classical conditioning *were introduced and tested (Ruf et al., 2013). Semantic classical conditioning refers to establishing a cortical response to the *trueness* of a statement (i.e., its semantics) irrespective of the particular constituent words and letters or sounds of the words. For this, an unconditioned stimulus (e.g., an acoustic signal or short electrical pulse) is presented only when a true statement is acoustically presented (conditioning and calibration of the classifier). After conditioning, cortical responses are evaluated online (e.g., using linear discriminant analysis) and classification results fed back to the subject at the end of each trial. In a first study involving healthy volunteers, a mean accuracy of 65.4% for classification of “yes” and 68.8% for “no” thinking was found. Offline analysis of the conditioned cortical responses (as measured by area under curve of 2 s-time intervals following acoustic presentation of a true or false statement) revealed significant differences between conditioned “yes” and “no” answers. Successful translation of this approach to LIS patients was later demonstrated in a clinical study that consisted of two weeks of measurements with four sessions per week (De Massari et al., 2013). Although online classification of brain responses was around chance level when averaged across all sessions, use of a non-linear classification algorithm (radial basis function kernel support vector machine) resulted in substantial improvements in classification accuracies. This suggests that, in principle, semantic classical conditioning of brain activity is feasible, but that online single-trial analysis of electric brain activity may not be robust enough to establish reliable communication in CLIS patients. After it was shown that less training is required to learn voluntary control of blood-oxygenation-level–dependent (BOLD) signals compared to EEG signals (e.g., only a few real-time fMRI sessions are required to achieve >80% BCI control accuracy) (Birbaumer et al., 2013, Sitaramet al., 2017), it seemed reasonable to attempt establishing the semantic conditioning approach in an fMRI or *f*NIRS-based paradigm. However, use of fMRI in patients with complete paralysis, who usually depend on artificial respiration, is often not feasible. The availability of *f*NIRS-BCI systems allowing for bedside evaluation of metabolic brain activity led to first experiments in which semantic classical conditioning was attempted for restoration of communication in a patient diagnosed with CLIS (Birbaumer et al., 2014). Later, also classification of sensorimotor and temporal cortical oxygenation and de-oxygenation following simple questions with known positive or negative answers (e.g., “Your name is Giulia”) was attempted (Gallegos-Ayala et al., 2014). In healthy volunteers, classification of NIRS-responses associated with simple “yes” and “no” answers was successfully demonstrated and did not require any specific conditioning of cortical responses (Abdalmalaket al., 2020, Rezazadeh Sereshkehet al., 2019, Taninoet al., 2015). Due to the limited number of CLIS patients and significant challenges to ascertain a sufficient level of alertness (Soekadar et al., 2013), it remains open whether and to what degree communication in CLIS can be restored. Thus, further studies are needed to proof applicability and usefulness of *f*NIRS-BCIs for restoration of communication in CLIS.

Given that patients with prolonged disorders of consciousness are often misdiagnosed (Wang et al., 2020), beyond CLIS, there is critical need for robust non-invasive bed-side tools to evaluate level of consciousness and for re-establishing communication. Besides their immediate impact on self-determination and quality of life, such systems may also trigger neural recovery as it was shown for BCIs in restoration of movement and accelerate emergence from minimally conscious state (EMCS).

### 3.2 Optical BCIs in restoration of movement

Besides restoration of communication, restoration of movement became the second major pillar in clinical BCI research (Soekadar et al., 2011a). In 2003, a first non-invasive EEG-BCI was presented that enabled a quadriplegic patient to control grasping motions through functional electrical stimulation activated by modulation of sensorimotor rhythms (SMR) (Pfurtscheller et al., 2003). SMR is an oscillatory idle rhythm (9–15 Hz) recordable over the sensorimotor cortex that becomes desynchronized during motor imagery, motor planning or execution. It was shown that repeated use of such SMR-BCI-controlled hand exoskeleton can lead to cortical neuroplasticity (Soekadar et al., 2011b) and functional motor recovery in chronic stroke patients with severe finger paralysis (Cerveraet al., 2018, Pichiorriet al., 2015, Ramos-Murguialday et al., 2013). Given that optical BCIs can also translate motor imagery-related modulations of brain activity into control commands of external devices (Sitaram et al., 2007), such systems were soon tested as possible upper- and lower limb rehabilitation tools for stroke patients (Khanet al., 2018, Reaet al., 2014, Schurholzet al., 2012). Similar to EEG- and MEG-BCI paradigms, motor imagery-based *f*NIRS-neurofeedback induced higher activation of the premotor cortex, which was accompanied by improved self-assessed kinesthetic motor imagery during real neurofeedback as compared to a within sham-feedback condition in healthy participants (Mihara et al., 2012). Repeated feedback training of motor imagery-related brain activity led to more focused brain activation compared to sham feedback training using both an *f*NIRS- (Kober et al., 2014) or *f*MRI-based system (Marins et al., 2015). Beyond replicating this finding, a first double-blinded randomized sham-controlled pilot study involving 20 hemiplegic stroke patients showed a specific improvement of hand and finger function after six *f*NIRS-based real-time feedback training sessions (Mihara et al., 2013). Recently, (Fujimoto et al., 2017) successfully demonstrated regulation of the supplementary motor area (SMA) without providing any instruction of motor imagery or other mental strategies. Although, mental strategies were not systematically assessed after the training and participants may have engaged in motor imagery, this study underlines the potential of *f*NIRS-based neurofeedback for modulating brain activity in motor regions. Such neurofeedback may also be used to support motor learning after stroke. In an effort to exploit the advantages of both, (Rieke et al., 2020) introduced a combined, sequential real-time fMRI and *f*NIRS BCI system aiming at enhancing motor learning after stroke. The patient first underwent three fMRI-neurofeedback sessions receiving feedback of the ipsilesional motor cortex activity while engaging in a wrist extension training. The fMRI sessions allowed for a more precise localization of the signal source informing the channel selection in subsequent *f*NIRS neurofeedback sessions. During these *f*NIRS-sessions, the patient additionally received neural-triggered functional electrical stimulation (FES) to assist in wrist movements while the wrist extension training was continued.

Besides upper limb movements, also successful regulation of swallowing-related motor regions (within the inferior frontal gyrus) was demonstrated which may prove beneficial to treat dysphagia in the future (Koberet al., 2015, Koberet al., 2018, Koberet al., 2019).

Whereas classification accuracy of motor imagery-related cortical activation ranged between 70% and 90% in healthy subjects (Coyleet al., 2004, Coyleet al., 2007, Honget al., 2015, Naseer and Hong, 2013, Naseer and Hong, 2015, Sitaramet al., 2005), classification accuracies tend to be lower in patient populations (Blokland et al., 2014). More studies are needed to investigate the mechanisms underlying learned self-regulation of metabolic brain activity and how BCI-related functional and structural neuroplasticity relates to specific clinical improvements (Soekadaret al., 2015a, Ushiba and Soekadar, 2016).

### 3.3 Optical real-time brain imaging in neuropsychiatric disorders

Besides neurorehabilitation, optical real-time brain imaging may also represent a promising tool to improve brain functions and ameliorate symptoms in neuropsychiatric disorders.

E.g., eight sessions of *f*NIRS-neurofeedback training of prefrontal cortex activation improved inhibitory control in a subclinical sample of highly impulsive adults. This corresponded to higher activation of the left dorsolateral prefrontal cortex (dlPFC) during a Go-Nogo-task after the neurofeedback training compared to a control group receiving electromyography (EMG)-biofeedback (Hudak et al., 2017). Children diagnosed with attention deficit and hyperactivity disorder (ADHD) underwent a similar training protocol. After 12 sessions, study participants showed a trend towards improved inhibitory control, which was accompanied by symptom reduction. However, control groups (EEG- and EMG-biofeedback) also showed marginal symptom reduction and no significant group effect was found. This is in line with data of a small randomized controlled trial reporting only unspecific improvements of reading abilities in children with ADHD after 15 sessions of neurofeedback (Blume et al., 2020). In patients with social anxiety disorder, 15 sessions of *f*NIRS neurofeedback training of prefrontal brain regions reduced social threat-related attention bias and improved social and general trait anxiety as well as depressive symptoms (Kimmig et al., 2019). Due to absence of a control group, results are only preliminary, however.

In addition, training protocols have been developed to enhance the effects of a facial-recognition training in autism (Liu et al., 2017), reduce auditory verbal hallucinations and improve cognitive functioning in schizophrenia (Gomeset al., 2018, Storchaket al., 2019) and modulate eating behavior (Percik et al., 2019). To date, mostly single-case studies were reported, however.

Successful classification of neutral and positive affective states was demonstrated (Trambaiolli et al., 2018), and it was shown that participants could increase asymmetric activation of the left dlPFC by affective engagement with a virtual agent (Aranyi et al., 2016). These fNIRS-based affective BCIs developed in healthy participants may pave the way for their future application in the treatment of mood disorders such as major depression. Moreover, the effectiveness of fNIRS-neurofeedback training for modulating activation of the orbitofrontal cortex (OFC) was demonstrated in a sham-controlled study involving 60 healthy participants. This brain region was shown to be related to cognitive flexibility and reward processing. Psychiatric disorders characterized by dysregulations in these domains and associated with OFC dysfunction could be targeted with this neurofeedback protocol in future (Li et al., 2019).

### 3.4 Optical real-time brain imaging for neuroenhancement and neuroergonomics

Next to clinical applications, *f*NIRS-neurofeedback has been investigated as a tool for cognitive enhancement and improving stress resilience and well-being in healthy people. Aiming to reduce stress in Japanese workers (Kotozaki et al., 2014), combined neurofeedback of the frontal pole with a heart rate variability (HRV) biofeedback training. After four weeks of training in the participants’ home environment, they found improvements in job-related stress measures and lower cortisol responses alongside increases in grey matter volume in brain regions implicated in stress response (hippocampus) and emotion control (orbitofrontal cortex). Unfortunately, the study design did not allow to disentangle the effects of neurofeedback from HRV biofeedback or control for unspecific effects of the training. Lai et al. (2015) investigated the effects of a one-session neurofeedback training of the frontal pole on attentional networks. They reported improved levels of performance as compared with a passive control task (counting), and similar performance as compared to mindfulness meditation. This study provides first evidence that neurofeedback training may have similar effects on attention as mindfulness practices. However, studies employing larger training regimens and follow-up measures are needed to corroborate this finding and to investigate whether neurofeedback is more efficient than mediation as hypothesized by the authors. Also, differences in the involved neural mechanisms need to be investigated.

Recently, (Xu et al., 2020) introduced an *f*NIRS-neurofeedback training based on frontoparietal connectivity, and demonstrated its feasibility to improve working memory and attention in healthy people. Such an approach may also turn out to be useful in the treatment of neuropsychiatric disorders characterized by deficits in these cognitive domains or to prevent cognitive decline.

Beyond direct feedback and control of brain activity related to various mental or emotional states, including levels of vigilance, attention and mental workload, detecting such states could be used for passive BCIs creating supportive environments in which feedback or task demands are optimally adapted to the mental state of the user (Gerjetset al., 2014, Mulleret al., 2008, Zander and Jatzev, 2012). By mitigating the consequences of excessive workload or allowing for strategy changes, safety, performance, effectiveness or motivation in human–machine interaction could be increased. Such neuroergonomic approaches use markers in brain activity to assess covert mental states, such as mental workload, independently of the reported subjective or measured overt performance of a human operator (Parasuraman & Wilson, 2008). Sudden performance declines that follow sustained periods of excessive or too low task demands or engagements could be predicted and avoided, an issue of importance, e.g., in neurorehabilitation or psychotherapeutic interventions. In this context, HRV was recently identified as a promising biomarker predicting decline in brain/neural control performance (Nann et al., 2021). Moreover, varying workload conditions have been shown to modulate activation in the dorsolateral and ventrolateral prefrontal cortex as measured by *f*NIRS (Ayazet al., 2013, Brunoet al., 2018, Schreppelet al., 2008). Accordingly, frontal oxygen dependent metabolism measured over prefrontal regions using *f*NIRS could be successfully used for the assessment of expertise development and the assessment of cognitive workload (Ayazet al., 2013, Ayazet al., 2012, Causse et al., 2017).

While recently published studies support the potential of *f*NIRS-neurofeedback for clinical and non-clinical applications, a recent systematic review concluded that sufficiently powered studies and large randomized controlled trials are still lacking and specificity of the reported effects remains to be demonstrated (Kohl et al., 2020). Also, shortcomings in reporting important information were identified. E.g., online signal processing methods and training success measures were not reported by some of the studies. Moreover, it is noteworthy that some of the above mentioned studies did not use concentrations of HbO or HHb as a feature to calculate the feedback signal but used measures of the optical density of one wavelength (Kotozaki et al., 2014) or the ratio of light intensities of two different wavelengths (Laiet al., 2015, Perciket al., 2019). While these features should, in theory, carry information of HbO concentrations, methodological studies are required to find out if this simplified approach is comparable in terms of signal quality and performance. Future studies will benefit from adopting more rigorous research and reporting practices as encouraged by a recent consensus paper (Ros et al., 2020) and methodological recommendations (Kohl et al., 2020).

### 3.5 Advancing optical real-time brain imaging towards broader clinical adoption

While optical real-time brain imaging is clearly more suitable for neurofeedback than BCI applications due to its signal features in the seconds range, it was recently suggested that designing paradigms that combine both brain/neural control of an assistive device and instrumental conditioning of brain activity (neurofeedback) may provide decisive advantages to increase the impact of intervention (e.g., fostering generalization of learned skills from the laboratory to real-life scenarios while triggering neuroplasticity and neural recovery) (Soekadar et al., 2019). Thus, combination of optical brain imaging with other modalities, such as EEG or optically pumped magnetometers (OPM) that exploits shared and complementary information may substantially increase robustness and performance (Fazliet al., 2015, Fazliet al., 2012, von Luhmann, 2017). By broadening the versatility and reliability of use, such *hybrid* systems could extend the scope of optical real-time brain imaging towards broader clinical adoption. Numerous studies have investigated feasibility, safety and reliability of such hybrid systems across different brain regions and functional domains (Fazliet al., 2012, Kooet al., 2015, Muller-Putzet al., 2015, Shinet al., 2018) paving the way for various future clinical applications (for an overview of such systems targeting the motor cortex, see table 1).

**Table 1:** List of studies investigating feasibility of hybrid brain-computer interfaces involving optical brain imaging for neurofeedback of motor cortex activation. Besides details on the signal features used for neurofeedback, also achieved classification accuracies are provided.

Study	Brain area	Features used for neuroeedback	Classification accuracy
(Fazli et al., 2012)	bilateral motor cortex	NIRS: mean of △HbO and HbR EEG: Power	83.10%
(Koo et al., 2015)	bilateral motor cortex	NIRS: △HbO EEG: power, variance	88% (self-paced)
(Yin et al., 2015)	bilateral motor cortex	NIRS: signals from HbR, HbO, HbT, HbO - HbR, light absorption (two wavelengths) EEG: power, instantaneous phase, instantaneous amplitude, instantaneous frequency	89%
(Buccino et al., 2016)	bilateral motor cortex	NIRS: mean and slope of △HbO and HbR EEG: power, variance	72.2% (right / left)
(Li et al., 2017)	bilateral motor cortex	NIRS: Initial dip of HbO and HbR EEG: coefficient of discrete wavelet transform	91.02% (right / left)
(Fu et al., 2017)	bilateral motor cortex	NIRS: signals from HbR, HbO EEG: power, instantaneous phase, instantaneous amplitude, instantaneous frequency	74% (hand clenching)

## 4 Conclusions and future outlook

Optical real-time brain imaging has demonstrated its clinical relevance and is currently tested in a variety of applications, e.g. in the treatment of stroke (Mihara et al., 2013), ADHD (Blumeet al., 2020, Hudaket al., 2017, Marxet al., 2014), social anxiety disorder (Kimmig et al., 2019), autism spectrum disorder (Liu et al., 2017) and schizophrenia (Storchak et al., 2019). Further, protocols that were successfully tested in healthy participants are now paving the way for clinical applications of optical real-time brain imaging in neuropsychiatric disorders (Aranyiet al., 2016, Liet al., 2019, Trambaiolliet al., 2018, Xu et al., 2020). Particularly brain functions related to the frontal lobes, such as executive functions or emotion regulation that are often affected in depression, schizophrenia or neurodegenerative disorders, might be promising targets for neurofeedback applications. To establish optical real-time brain imaging as a robust and powerful clinical tool, a number of challenges related to the consistency of findings (Bendall et al., 2016) and possible confounds limiting specificity of *f*NIRS recordings (e.g., extracranial artifacts such as temporal muscle activity) have to be mastered, however (Schecklmann et al., 2017).

Besides further clinical validation, broader use of optical neurofeedback/BCI applications in and outside of the clinical context will also depend on further miniaturization, improvements in optical sensor technology and user-friendliness (von Lühmann et al., 2015). Increasing the performance of such systems, e.g., by further advancing hybrid BCIs or using innovative imaging techniques, will be critical to establish optical measures of brain activity in the context of assistive applications.

The particular potential in *hybrid* optical neurofeedback and BCI applications relates to its capability to incorporate an increasing number of bio-signal modalities and integration of contextual (environmental) information. In this regard, combination of such advanced context-sensitive systems with devices providing multi-sensory feedback to the user (e.g., comprising augmented or virtual reality, AR/VR, vibro-tactile/acoustic or haptic as well as heat and cold stimulation) are of special interest for the medical field. Not only might such systems improve *assistive* brain/neural control (Crea et al., 2018, Nannet al., 2020), but such multi-level closed-loop systems might also prove particularly effective and useful to purposefully modulate the brain’s network activity in the context of neurofeedback applications.

By focusing on the use of both electrical/magnetic and bio-optical signals from both brain and body, and exploiting the advantages of each modality and signal, the design of more comprehensive and robust human–machine interfaces becomes possible: Toward unobtrusive, hazard free applications with high usability that can be reliably integrated into the user’s daily life.

Successful development of such multi-modal neurofeedback/BCI systems will be the result of highly interdisciplinary collaborations that combine understanding of physiology, clinical conditions and symptoms, instrumentation, application, and last but not least methodology.

In analogy to clinical neurofeedback/BCI applications based on other modalities, broad use and implementation of optical real-time brain imaging in everyday life environments raise a number of important neuroethical questions, such as privacy, data security or accessibility, that need to be considered (Clausen et al., 2017). This is particularly true for applications in neuroenhancement or neuroergonomics that serve other purposes than medical applications aiming at restoration of lost or compromised brain or body function.

## Footnotes

1 For a more comprehensive overview of *f*NIRS-neurofeedback studies, please see Kohl et al. (2020) as well as https://osf.io/hnxfq/ providing a comprehensive database that includes methodological details of published studies
